# Supplementary material for: MetaQTL: a package of new computational methods for the meta-analysis of QTL mapping experiments
Source: BMC Bioinformatics. 2007 Feb 8;8:49. doi: 10.1186/1471-2105-8-49 (PMC1808479; doi:10.1186/1471-2105-8-49)
Supplement: Additional File 7 — MetaQTL Package : jar file and tutorial. This Zip archive contains both the MetaQTL JAR file and the files of the tutorial. [file 1471-2105-8-49-S7.zip › org.inra.metaqtl/doc/org/metaqtl/algo/QtlTreeAlgorithm.html]

QtlTreeAlgorithm


|  |  |  |
| --- | --- | --- |
| **Overview** \| **Package** \| **Class** \| **Use** \| **Tree** \| **Deprecated** \| **Index** \| **Help** | |  |
| **PREV CLASS**   **NEXT CLASS** | **FRAMES**    **NO FRAMES**     **All Classes** |  |
| SUMMARY: NESTED \| FIELD \| CONSTR \| METHOD | DETAIL: FIELD \| CONSTR \| METHOD |  |


---


## org.metaqtl.algo Class QtlTreeAlgorithm

```
java.lang.Object
  org.metaqtl.algo.MetaAlgorithm
      org.metaqtl.algo.ClustAlgorithm
          org.metaqtl.algo.QtlTreeAlgorithm
```

**All Implemented Interfaces:**: IMetaAlgorithm

---

``` public class QtlTreeAlgorithm extends ClustAlgorithm ```

This class defines methods to perform agglomerative
hierarchical QTL clustering.

---

| **Field Summary** | |
| --- | --- |
| `int` | `tree_method`             This global variable defines the method used by the aglomerative hierarchical clustering. |

**Fields inherited from class org.metaqtl.algo.ClustAlgorithm**

`chrName, chromosomes, missing_sd_mode, ontology, result, sd_mode`

**Fields inherited from class org.metaqtl.algo.MetaAlgorithm**

`logger, loggerUp, workAmount, workProgress`


**Constructor Summary**

`QtlTreeAlgorithm(IBioGenome map,
IBioOntology ontology)`


| **Method Summary** | |
| --- | --- |
| `MetaQtlResult[]` | `buildQtlTree(MetaQtlData data)`             For the given data points this method build the tree which represents the hierarchical clustering. |
| `void` | `run()`             This method performs an agglomerative hierarchical QTL clustering on each chromosome and returns the result as a `MetaQtlAnalysis` object. |
| `void` | `setTreeMethod(int tree_method)` |

**Methods inherited from class org.metaqtl.algo.ClustAlgorithm**

`getResult, setCIMiss, setCIMode`

**Methods inherited from class org.metaqtl.algo.MetaAlgorithm**

`disableLogger, getLogger, getWorkAmount, getWorkProgress, isLoggerEnable, setLogger, setLogger`

**Methods inherited from class java.lang.Object**

`clone, equals, finalize, getClass, hashCode, notify, notifyAll, toString, wait, wait, wait`

**Field Detail**

### tree\_method

```
public int tree_method
```

:   This global variable defines the method used by the aglomerative
    hierarchical clustering.


**Constructor Detail**

### QtlTreeAlgorithm

```
public QtlTreeAlgorithm(IBioGenome map,
                        IBioOntology ontology)
```


**Method Detail**

### run

```
public void run()
```

:   This method performs an agglomerative hierarchical QTL clustering
    on each chromosome and returns the result as a `MetaQtlAnalysis`
    object.
    To control the behaviour of the algorithm use the global variables
    of the class.

    :   **Parameters:**: `map` -

---


### buildQtlTree

```
public MetaQtlResult[] buildQtlTree(MetaQtlData data)
```

:   For the given data points this method build the tree which
    represents the hierarchical clustering.

    :   **Parameters:**: `data` - the QTK data points. **Returns:**: the results of the hierarchical clustering. **See Also:**: `HClustAlgorithm`

---


### setTreeMethod

```
public void setTreeMethod(int tree_method)
```

:   **Parameters:**: `centroid_method` -


---


|  |  |  |
| --- | --- | --- |
| **Overview** \| **Package** \| **Class** \| **Use** \| **Tree** \| **Deprecated** \| **Index** \| **Help** | |  |
| **PREV CLASS**   **NEXT CLASS** | **FRAMES**    **NO FRAMES**     **All Classes** |  |
| SUMMARY: NESTED \| FIELD \| CONSTR \| METHOD | DETAIL: FIELD \| CONSTR \| METHOD |  |


---
